# Supplementary material for: tRF-BERT: A transformative approach to aspect-based sentiment analysis in the bengali language
Source: PLoS One. 2024 Sep 20;19(9):e0308050. doi: 10.1371/journal.pone.0308050 (PMC11414928; doi:10.1371/journal.pone.0308050)
Supplement: Attachment — Submitted filename: tRF_BERT_Response to Reviewers.pdf [file pone.0308050.s001.pdf]

Journal: PLOS ONE
Manuscript ID: PONE-D-24-01459
Title: tRF-BERT: A transformative approach to aspect-based sentiment analysis in the bengali language
Authors: Shihab Ahmed, Moythry Manir Samia, Maksuda Haider Sayma, Md Mohsin Kabir, M. F. Mridha

Dear Editor and Reviewers,

The authors thank all reviewers and the editor for their acceptance of revising our paper and for their specific and essential comments. We have revised the paper and restructured several sections. The updated version presents all the changes.

Response of Editor

Editor Comment-1: Given the journal's biomedical and public health focus, some applications of sentiment analysis in public health research should be highlighted in the Introduction section (see: https://pubmed.ncbi.nlm.nih.gov/37376407; https://pubmed.ncbi.nlm.nih.gov/37358808).

Author's Response: We thank the Editor for suggesting these studies for improving our paper.
Author's Action: To address the editor's concern, two new paragraphs were added at the beginning of the "Introduction" section, along with the recommended studies as examples.

Editor Comment-2: Although the authors provided a description of the proposed hybrid transformative Random Forest and Bidirectional Encoder Representations from Transformers (tRF-BERT) model, there is limited explanation on the technical underpinnings, such as the specific architecture details, the interaction between Random Forest and BERT components, and how exactly the hybrid model outperforms its constituent parts.

Author's Response: We appreciate the Editor for emphasizing this crucial aspect to enhance our paper.
Author's Action: Architectural details of both BERT and Random Forest are provided in the "BERT model" and "RF" subsections of the "Methods and Materials" section of the manuscript. Also check Figs 2, 3, 4 in the manuscript. Additionally, the specifics of the proposed tRF-BERT model are presented in the "Proposed tRF-BERT Model" subsection. Only the newly added segments are highlighted in blue in the manuscript.

Editor Comment-3: While the manuscript mentions using two open-source benchmark datasets, Cricket and Restaurant, for Aspect-Based Sentiment Analysis, I am unable to find any citations for these datasets, and the authors do not provide further statistics about these datasets (e.g., number of samples, distri-

bution of classes). A more thorough dataset description is necessary.

Author's Response: We express our gratitude to the Editor for emphasizing the importance of this aspect in enhancing our paper.
Author's Action: The citations for the datasets and the link to the data source are included in the "Data Availability" section of the manuscript. Additional statistical information regarding the datasets, such as the number of samples and class distribution, has been included in the "Data source" subsection within the "Data collection and preprocessing" section in Tables 2 and 3 in the manuscript.

Editor Comment-4: The comparison against existing works primarily focuses on the final performance metrics. A comparison which discusses the nature of the datasets used in those works, model complexities, and computational resources required, would offer a clearer picture of the proposed model's advantages and limitations.

Author's Response: We appreciate the Editor's helpful suggestion.

The comparison between related works and ours is presented in Table 14 in the manuscript. All the studies discussed in Table 14 use the same two datasets as we did. The statistics related to these two datasets are provided in Tables 2 and 3 in the manuscript.

Editor Comment-5: The author strongly focuses on F1 score and accuracy for evaluating model performance. Incorporating additional metrics such as Precision-Recall AUC, Matthews Correlation Coefficient, or analysis on the model's performance across different aspects/categories could provide a more comprehensive evaluation.

Author's Response: We thank the Editor for this observation. We are especially grateful to the editor for suggesting additional metrics such as Precision-Recall AUC and Matthews Correlation Coefficient, as well as recommending analysis of the model's performance across different aspects/categories. We intend to include these suggestions in future editions.
Author's Action: We incorporated accuracy, precision, recall, and F1-score as evaluation metrics, which helped us evaluate the models and compare them with others in the domain. The response to this comment can be found in the "Performance Evaluation" subsection in "Methods and materials" section and "Result Analysis" section of the manuscript.

Editor Comment-6: While some hyperparameters are listed, the process of selecting these values or any optimization strategy employed is not discussed. Detailing the hyperparameter tuning process, including the range of values explored, would strengthen the methodological rigor.

Author's Response: We thank the editor for pointing out this important point.
Author's Action: We have tried our best to address this issue in our manuscript in the subsection "Hyperparameters" of the "Methods and Materials" section. The first paragarph has been added to the manuscript and colored blue as a response to this comment.

Response of Reviewer-1

Reviewer-1 Comment-1: In the abstract section, there is a sentence "it was clear that all the models used for our work achieved better results than any of the previous work", what does the "it" word refer to? If it is not unclear, please revise!

Author's Response: We thank the reviewer for the observation.

Author's Action: We revised the mentioned sentence in the "Abstract" section for better understanding.

Reviewer-1 Comment-2: In the sentence "A crucial part of this research involved finding or creating a dataset specifically designed for Bengali aspect-level sentiment analysis" on page 2, the authors mention that this paper creating a dataset. However, the page 7 mentioned that this study used publicly available datasets. Please clarify this issue!

Author's Response: We thank the reviewer for mentioning the issue. In our research work, we didn't create a new dataset; instead, we used publicly available datasets.

Author's Action: We changed the sentence mentioned in the "Introduction" section making the matter clear.

Reviewer-1 Comment-3: There is the sentence "it's crucial to consider the semantics of the given aspect as a new and distinct piece of information, separate from the context itself" on page 4. Do not use abbreviations in academic paper such as "it's" and do not use the "it" word for syntactic expletive in academic papers. Use this concept consistently in the whole paper!

Author's Response: We thank the reviewer for making this suggestion regarding our paper.

Author's Action: We updated the mentioned sentence according to the reviewer's advice in the first paragraph of the "Literature Review" section.

Reviewer-1 Comment-4: What is used pre-trained BERT and RoBERTa models for fine-tuning process? Did authors perform a pre-training process for the BERT model or use already pre-trained models by others? The author should clearly mention this issue.

Author's Response: We thank the reviewer for mentioning the issue. For this study, we used already pre-trained BERT and RoBERTa models by others.

Author's Action: Please refer to the first paragraph with the heading "BERT-based model" in the "Proposed tRF-BERT model" subsection in the "Methods and materials" section of the manuscript.

Reviewer-1 Comment-5: In Tokenization and embeddings section on page 8, the paper mentioned the use of 'bert-base-uncased' tokenizers for tokenization. However, 'bert-base-uncased' and 'roberta-base' tokenizers are pre-trained in English. How these tokenizers can be implemented in Bengali language?

Author's Response: We thank the reviewer for focusing on this issue.
Author's Action: A short explanation of using 'bert-base-uncased' and 'roberta-base' tokenizers for our Bengali datasets is added to the manuscript. We added this explaination in "Tokenization and embeddings" subsection of "Methods and materials" section of our manuscript.

Reviewer-1 Comment-6: What are M, N, and T terms in the BERT model? Please define them!

Author's Response: We thank the reviewer for the observation.
Author's Action: We briefly defined M, N, and T in "BERT model" sub-subsection of "Aspect and sentiment prediction models" subsection of "Methods and materials" section.

Reviewer-1 Comment-7: In the BERT model section on page 10, please briefly define these unique tokens i.e., [CLS], [SEP], and [EOS]!

Author's Response: We thank the reviewer for the observation.
Author's Action: We briefly defined the unique tokens i.e., [CLS], [SEP], and [EOS] in "BERT model" sub-subsection of "Aspect and sentiment prediction models" subsection of "Methods and materials" section.

Reviewer-1 Comment-8: If the authors want to elaborate Q, K, and V parameters, how do these parameters come and the correlation with the BERT inputs, i.e., X and Y?

Author's Response: We thank the reviewer for the observation.
Author's Action: We elaborated on Q, K and V parameters and their correlation with BERT inputs in "BERT model" sub-subsection of "Aspect and sentiment prediction models" subsection of "Methods and materials" section.

Reviewer-1 Comment-9: Please paraphrase this sentence "It is marked as "IsNext" if it does, and "NotNext" if it doesn't." What does "it" refer to?

Author's Response: We appreciate the reviewer's helpful suggestion. The word "it" refers to the relationship between the second sentence and the first sentence in the original text.
Author's Action: However, we paraphrased the sentence for better understanding in the "BERT model" sub-subsection of the "Aspect and sentiment prediction models" subsection of "Methods and materials" section.

Reviewer-1 Comment-10: As we can see in Table 2 and 3, the number of each data class or category is imbalanced, how the proposed model can address this issue?

Author's Response: We thank the reviewer for mentioning the issue. To evaluate the performance of our model, we employed a combination of metrics: Accuracy, Precision, Recall, and F1-Score. While Accuracy is sufficient for balanced datasets, imbalanced datasets require a more nuanced approach. In such cases, Precision, Recall, and F1-Score provide valuable insights. By considering these metrics, we gain a comprehensive understanding of how well our model identifies every case, even when the classes are not evenly distributed.

Author's Action: As shown in Tables 7, 8, 9 and 10 in the manuscript, our proposed model demonstrates strong performance across all evaluation metrics, indicating its effectiveness in handling both balanced and imbalanced scenarios. A paragraph addressing this issue is added in the "Results" subsection right above Table 7 of the "Result analysis" section in the manuscript.
In future work, we aim to address the issue of imbalanced data classes by exploring various data balancing techniques such as oversampling, undersampling, and class weighting.

Reviewer-1 Comment-11: Are Bangla and Bengali different terms? If they are the same term, please choose one of them and use consistently the selected term.

Author's Response: We thank the reviewer for pointing out this important point. Bengali is the same term as Bangla. Bangla is the language's name in Bangla, while Bengali is the term used in English.
Author's Action: To avoid confusion, we used "Bengali" consistently throughout the paper.

Reviewer-1 Comment-12: In the performance evaluation, the proposed model used cross-validation, please provide more explanation in the implementation such as what is the value of the k parameter.

Author's Response: We appreciate the esteemed reviewer for highlighting this concern. Author's Action: In response, we have included the value of K in the "Cross-Validation" subsection of the "Methods and materials" section of the manuscript. Only the newly added lines are highlighted in blue.

Reviewer-1 Comment-13: In the experimental result of aspect detection and sentiment classification in this research, please also include the results if the model only uses TF-IDF and RF for the classification task to be presented in Tables 7-10. How is the performance of TF-IDF and RF?

Author response: We are grateful to the respected reviewer for the valuable comments. Author's Action: We have included the results obtained from the Random Forest model with TF-IDF in Tables 7, 8, 9, and 10 in the manuscript.

Reviewer-1 Comment-14: The authors can cite this paper https://doi.org/10.1186/s40537-023-00782-9 that also proposed hybrid strategy for sentiment analysis.

Author's Response: We thank the reviewer for suggesting this supporting material for our study. Author's Action: We have cited this study in the seventh paragraph in "Introduction" section of the manuscript.

Response of Reviewer-2

Reviewer 2 Comment-1:This paper proposes a mix of random forest and pre-trained transformer model for two Aspect Based Sentiment Analysis (ABSA) tasks: aspect category detection and aspect sentiment classification. This study has been primarily targeted towards Bengali language and it used two existing Bengali text data in their experiments. The results show that the proposed tRF-BERT model outperforms ABSA tasks compared to tasks done with independent models.

Pros: 1. ABSA for low resource languages like Bengali is interesting 2. The proposed model looks like an ensemble model for ABSA tasks, which is interesting 3. Focus on two different tasks is a plus

Author response: We thank the respectful reviewer for these excellent remarks.

Reviewer 2 comment-2: Cons: 1. There is no novelty in this paper. The only novel part in this paper is combining results of two different classifiers and feeding them to a neural network model for final classification. However, this looks like an extended random forest algorithm and it just gets more data for a classification task.

Author's Response: We thank the reviewer for providing such insightful observations. We have tried our best to respond.
Author's Action: Our proposed model outperformed all existing works on the publicly available 'Cricket' and 'Restaurant' datasets in the field of Bengali ABSA. The contributions made by our study are the markers that ensure the novelty of the research in the field of Bengali ABSA. The contributions made by our study are mentioned in the "Introduction" section of the manuscript.

Reviewer 2 Comment-3:There are several questions with experiments: (a) Why the proposed model has not been evaluated against other ABSA models? This area is fast growing research and many methods can be applied approximately to other languages too. (b) Why results of ABSA tasks just with random forest are not given? (c) I couldn't find any citations for the dataset (d) Why the runtime is not compared? Is this performance boost worth the execution time this model takes? (e) Why no experiments done on English datasets?

Author's Response: We are grateful to our respected reviewer for the valuable comments.

(a) Author's Action: Our research primarily concentrated on Bengali Aspect-Based Sentiment Analysis (ABSA) tasks. As such, our investigation was limited to comparing our model exclusively with existing Bengali ABSA models. The comparison with existing Bengali ABSA models is given in Table 14 in our manuscript in the "Comparison with previous works" subsection. However, we aim to extend our evaluation in future studies to include comparisons with models designed for various other languages.

(b) Author's Action: We have included the results obtained from the Random Forest

model with TF-IDF in Tables 7, 8, 9, and 10 in the manuscript.

(c) Author's Action: The datasets used in this study are available at [https://github.com/atik-05/Bangla_ABSA_Datasets] Additionally, citation 16 in the reference section provides further details about the dataset and includes the DOI link.

(d) Author's Action: We have incorporated a comparison of runtime in Table 11 in the manuscript in the "Results" subsection in the "Result and analysis" section.

(e) Author's Action: In our research, we specifically focused on only the Bengali Dataset. In the future, we plan to extend our work to English and others languages datasets.

Reviewer 2 Comment-4: The random forest model uses TF-IDF features. This ignores the concept of language models and makes them inefficient and unreliable for ABSA tasks. For TF-IDF features, the model should know all the data beforehand, including some of the words from the test data. How this method can scale for larger problems?

Author Response: We appreciate the valuable feedback from our esteemed reviewer. Author's Action: While experimenting with approaches like BERT Tokenizer and Word2Vec, we found that for our specific datasets, TF-IDF yielded better results in our hybrid model. The accuracy of each approach for tRF-BERT is given in Table 12 in the "Results" subsection of the "Result and analysis" section of the manuscript. Also, we provided the probable reason for such results after Table 12 in the manuscript. Newly added sections are highlighted in blue.

Reviewer 2 Comment-5:Is it not possible to add other models like SVM, CNN, or other traditional models. How the performance change in that scenario?

Author response: We appreciate this insightful comment.
Author's Action: We experimented with other models like Logistic Regression (LR), and SVM for building our model. We found that the combination of BERT and RF work best compared to the others. Results of the experiments are given in Table 13 in our manuscript in the "Results" subsection of the "Result and analysis" section.

Reviewer-2 Comment-6: It is evident from previous methods that random forest perform very poorly in ABSA tasks. How does it make sense to give equal importance to both model predictions? Can it be weighted?

Author response: We appreciate this insightful comment.
Author's Action: The response to this comment is added in the "Aspect and sentiment prediction models" subsection in the "Methods and materials" section of the manuscript. The newly added paragraphs are highlighted in blue after the "Proposed tRF-BERT model" subsection in the manuscript.

Reviewer-2 Comment-7: I do not understand how the predictions are fed again into a neural network

Author response: We appreciate this insightful comment. The predictions from both the BERT and Random Forest models are combined into a single feature vector, termed 'combined_features', which serves as the input to a neural network. This neural network comprises layers with ReLU activation for nonlinear transformations, dropout to prevent overfitting, and softmax activation for predicting polarity class probabilities.

Author's Action: The response to this comment is available in the "Proposed tRF-BERT Model" subsection of the "Methods and materials" section of the manuscript.
