# Supplementary material for: tRF-BERT: A transformative approach to aspect-based sentiment analysis in the bengali language
Source: PLoS One. 2024 Sep 20;19(9):e0308050. doi: 10.1371/journal.pone.0308050 (PMC11414928; doi:10.1371/journal.pone.0308050)
Supplement: Attachment — Submitted filename: tRF_BERT.pdf [file pone.0308050.s002.pdf]

Journal: PLOS ONE
Manuscript ID: PONE-D-24-01459
Title: tRF-BERT: A transformative approach to aspect-based sentiment analysis in the bengali language
Authors: Shihab Ahmed, Moythry Manir Samia, Maksuda Haider Sayma, Md Mohsin Kabir, M. F. Mridha

Dear Editor and Reviewers,

The authors thank all reviewers and the editor for their acceptance of revising our paper and for their specific and essential comments. We have revised the paper and restructured several sections. The updated version presents all the changes.

Response of Reviewer-1

Reviewer-1 Comment-1: The authors generally already addressed my previous comments. However, there is a point that should be clarified by the author as following: - The authors said that they used "pre-trained BERT and RoBERTa models by others". Please give the reference where those pre-trained models can be found.

Author's Response: We thank the reviewer for pointing out the issues.
Author's Action: We've already referenced two papers in Citation 14 and 32, providing in-depth insights into pre-trained models initially, though not uniformly. Now, these references are consistently cited throughout the text wherever pre-trained models are mentioned.
