# Supplementary material for: tRF-BERT: A transformative approach to aspect-based sentiment analysis in the bengali language
Source: PLoS One. 2024 Sep 20;19(9):e0308050. doi: 10.1371/journal.pone.0308050 (PMC11414928; doi:10.1371/journal.pone.0308050)
Supplement: Attachment — Submitted filename: Response to Reviewers (1).docx [file pone.0308050.s003.docx]

Journal: PLOS ONE

Manuscript ID: PONE-D-24-01459

Title: tRF-BERT: A transformative approach to aspect-based sentiment analysis in the bengali language

Authors: Shihab Ahmed, Moythry Manir Samia, Maksuda Haider Sayma, Md Mohsin Kabir, M. F. Mridha

Dear Editor and Reviewers,

The authors thank all reviewers and the editor for their acceptance of revising our paper and for their specific and essential comments. We have revised the paper and restructured several sections. The updated version presents all the changes.

Response of Reviewer-2

**Reviewer-2 Comment-1: Authors have addressed most of the previous reviews. However, I have the following comments for this revised manuscript:**

**Use of TF-IDF features in the proposed model: TF-IDF performs decent, not only in the ABSA problem, but also for several other NLP classifiers. The only problem with TF-IDF is its lack of generalization. Authors did not show any evidence how the model performs if tokens are not present during the training phase but becomes available during the test. How about other classifier models like SVM and NB instead of Random Forest? They are traditional ML models too.**

Author’s Response: We thank the reviewer for pointing out the issues. We have tried our best to make the use of the tokenizer during the training phase more explicit.

Author’s Action: It is known that TF-IDF works well with trained words or vocabularies. In our research, we used vocabularies that are most commonly used in sentiment analysis, and as a result, TF-IDF performed satisfactorily. For future work, we intend to explore other technologies such as word2vec and BERT tokenizers to address the issue of generalization and improve the model’s performance with previously unseen tokens.

The tokens are present during the training phase, as shown in Figure 4 on page 17 (Section “Methods and Materials”). We have also added a statement indicating the use of the tok- enizer with TF-IDF in the last paragraph of the subsection “Tokenization and Embeddings” in the “Methods and Materials” section. (New additions are coloured blue.)

Additionally, we have experimented with SVM and NB instead of Random Forest with the BERT model, and the results are mentioned in Table 13 on page 22. To avoid confusion, we have updated the names of the models.

1
